# Treatment of Hypercalcemic Hyperparathyroidism After Kidney Transplantation Is Associated With Improved Allograft Survival

**DOI:** 10.1093/oncolo/oyad314

**Published:** 2023-11-24

**Authors:** Rongzhi Wang, Rhiannon D Reed, Griffin Price, Peter Abraham, Marshall Lewis, Jessica Liu McMullin, Paul MacLennan, Cozette Killian, Jayme E Locke, Song Ong, Vineeta Kumar, Andrea Gillis, Brenessa Lindeman, Herbert Chen, Jessica Fazendin

**Affiliations:** Department of Surgery, University of Alabama at Birmingham Heersink School of Medicine, Birmingham, AL, USA; Department of Surgery, University of Alabama at Birmingham Heersink School of Medicine, Birmingham, AL, USA; School of Medicine, University of Alabama at Birmingham Heersink School of Medicine, Birmingham, AL, USA; Department of Surgery, University of Alabama at Birmingham Heersink School of Medicine, Birmingham, AL, USA; School of Medicine, University of Alabama at Birmingham Heersink School of Medicine, Birmingham, AL, USA; Department of Surgery, University of Alabama at Birmingham Heersink School of Medicine, Birmingham, AL, USA; Department of Surgery, University of Alabama at Birmingham Heersink School of Medicine, Birmingham, AL, USA; Department of Surgery, University of Alabama at Birmingham Heersink School of Medicine, Birmingham, AL, USA; Department of Surgery, University of Alabama at Birmingham Heersink School of Medicine, Birmingham, AL, USA; Department of Medicine, University of Alabama at Birmingham Heersink School of Medicine, Birmingham, AL, USA; Department of Medicine, University of Alabama at Birmingham Heersink School of Medicine, Birmingham, AL, USA; Department of Surgery, University of Alabama at Birmingham Heersink School of Medicine, Birmingham, AL, USA; Department of Surgery, University of Alabama at Birmingham Heersink School of Medicine, Birmingham, AL, USA; Department of Surgery, University of Alabama at Birmingham Heersink School of Medicine, Birmingham, AL, USA; Department of Surgery, University of Alabama at Birmingham Heersink School of Medicine, Birmingham, AL, USA

**Keywords:** hyperparathyroidism, hypercalcemia, kidney transplant, allograft function

## Abstract

**Background:**

Hyperparathyroidism (HPT) and malignancy are the most common causes of hypercalcemia. Among kidney transplant (KT) recipients, hypercalcemia is mostly caused by tertiary HPT. Persistent tertiary HPT after KT is associated with allograft failure. Previous studies on managing tHPT were subjected to survivor treatment selection bias; as such, the impact of tertiary HPT treatment on allograft function remained unclear. We aim to assess the association between hypercalcemic tertiary HPT treatment and kidney allograft survival.

**Materials and Methods:**

We identified 280 KT recipients (2015-2019) with elevated post-KT adjusted serum calcium and parathyroid hormone (PTH). KT recipients were characterized by treatment: cinacalcet, parathyroidectomy, or no treatment. Time-varying Cox regression with delayed entry at the time of first elevated post-KT calcium was conducted, and death-censored and all-cause allograft failure were compared by treatment groups.

**Results:**

Of the 280 recipients with tHPT, 49 underwent PTx, and 98 received cinacalcet. The median time from KT to first elevated calcium was 1 month (IQR: 0-4). The median time from first elevated calcium to receiving cinacalcet and parathyroidectomy was 0(IQR: 0-3) and 13(IQR: 8-23) months, respectively. KT recipients with no treatment had shorter dialysis vintage (*P* = .017) and lower PTH at KT (*P* = .002), later onset of hypercalcemia post-KT (*P* < .001). Treatment with PTx (adjusted hazard ratio (aHR) = 0.18, 95%CI 0.04-0.76, *P* = .02) or cinacalcet (aHR = 0.14, 95%CI 0.004-0.47, *P* = .002) was associated with lower risk of death-censored allograft failure. Moreover, receipt of PTx (aHR = 0.28, 95%CI 0.12-0.66, *P* < .001) or cinacalcet (aHR = 0.38, 95%CI 0.22-0.66, *P* < .001) was associated with lower risk of all-cause allograft failure.

**Conclusions:**

This study demonstrates that treatment of hypercalcemic tertiary HPT post-KT is associated with improved allograft survival. Although these findings are not specific to hypercalcemia of malignancy, they do demonstrate the negative impact of hypercalcemic tertiary HPT on kidney function. Hypercalcemic HPT should be screened and aggressively treated post-KT.

## Teaser

The effect of tertiary hyperparathyroidism treatment on allograft function is unclear. This article assesses the association between hypercalcemic tertiary hyperparathyroidism treatment and kidney allograft survival.

**Implications for Practice**

To the authors’ knowledge, this is the first study to leverage time-varying survival analyses to overcome survivor treatment selection bias in the treatment of tertiary hyperparathyroidism and to clearly demonstrate an association between treatment of tertiary hyperparathyroidism and improved allograft survival. With an exponential increase in the need for kidney transplant worldwide and vastly insufficient kidney donors, this study provides evidence that hyperparathyroidism should be screened and treated following kidney transplant to achieve this survival benefit and protect a finite public health resource.

## Introduction

Hypercalcemia is relatively uncommon in the general population, with an overall prevalence of 1 in 1000 individuals. The most common causes of hypercalcemia are malignancy and hyperparathyroidism (HPT).^1,2^ There are 4 mechanisms of malignancy-related hypercalcemia, including humoral hypercalcemia via the secretion of parathyroid hormone-related peptide (PTHrP), local osteolytic hypercalcemia, excess production of extra-renal 1,25-dihydroxyvitamin D, and ectopic secretion of parathyroid hormone.^3^ The primary goal of therapy is the treatment of the underlying malignancy. Antihypercalcemia therapy should be considered an interim measure.^4^ The available treatments for malignancy-related hypercalcemia include intravenous hydration, calcitonin, bisphosphonates, corticosteroids, denosumab, and calcimimetics.^3^

HPT is caused by overactive parathyroid glands and can be primary, secondary, or tertiary. In primary hyperparathyroidism, one or more parathyroid glands secrete excess parathyroid hormone, resulting in hypercalcemia without a known stimulus.^5^ Parathyroidectomy (PTx) is the only curative treatment for primary hyperparathyroidism.^6^ Secondary HPT is nearly universal in patients with end-stage kidney disease (ESKD).^7^ PTH is elevated in response to decreased production of calcitriol from failed kidneys, elevated serum phosphorus, and decreased serum calcium.^8^ The Kidney Disease Improving Global Outcomes (KDIGO) guideline recommends maintaining PTH levels in the range of 2 to 9 times the upper normal limit in patients with ESKD.^9^ Treatment of secondary HPT usually includes PTx and the use of calcimimetics, such as cinacalcet. Successful kidney transplantation (KT) corrects the endocrine and metabolic imbalances responsible for secondary HPT.^10^ However, secondary HPT can persist and become tertiary HPT post-KT.^11^ Tertiary HPT is caused by autonomous function of multiple hyperplastic parathyroid glands, characterized by elevated parathyroid hormone (PTH) levels with hypercalcemia or normocalcemia.^12^Persistent HPT post-KT carries a significant clinical burden and is underdiagnosed and undertreated.^13^ The reported prevalence of tertiary HPT post-KT can be as high as 80%.^14^ Tertiary HPT is associated with an increased risk of cardiovascular events, renal osteodystrophy, pathologic fractures, graft loss, and mortality in KT recipients.^15-17^

Despite the detrimental clinical consequences of tertiary HPT, the guidelines for managing tertiary HPT post-KT are unclear. KDIGO guidelines suggested treatment with vitamin D analogs in the first 12 months post-KT.^9^ However, the use of vitamin D analogs is often limited by concurrent hypercalcemia.^12^The American Association of Endocrine Surgeons (AAES) guidelines recommend PTx as the treatment of choice for KT recipients with hypercalcemic tertiary hyperparathyroidism, with referral for PTx within 12 months post-KT.^12^PTx is the only curative treatment for HPT.^18-21^ Calcimimetics offer an alternative to PTx by acting as allosteric activators of calcium-sensing receptor in the parathyroid glands and other tissues to suppress PTH secretion.^8^ Cinacalcet, a calcimimetic, has been approved in the US since 2004 for dialysis patients with secondary HPT. While not approved for treatment of tertiary HPT, cinacalcet is also used for KT recipients with hypercalcemia.^12^

The treatment of hypercalcemic tertiary HPT with regard to allograft function remains controversial.^22^ Several studies reported a decline in allograft function following PTx^10,23-26^ or the use of cinacalcet.^27^ However, other studies found no changes in allograft function following PTx^8,23,28,29^ or cinacalcet.^30^ The discrepancy is partly due to variation in length of follow-up and a lack of a control group to adjust the confounding effect of tertiary HPT itself. Finnerty et al. concluded that PTx was associated with a lesser rate of allograft failure than cinacalcet in KT recipients.^31^ However, the study was prone to survivor treatment selection bias.^32^ The question remains whether the benefit of improved allograft survival is due to immortal bias (ie, the benefit of PTx itself, or the fact that KT recipients who live longer have more opportunities to select PTx). Therefore, we aimed to evaluate the relationship between treatment for tertiary HPT and long-term allograft survival, mitigating survivor treatment selection bias by utilizing treatment of tertiary HPT as a time-varying variable.

## Materials and Methods

**Figure 1.**
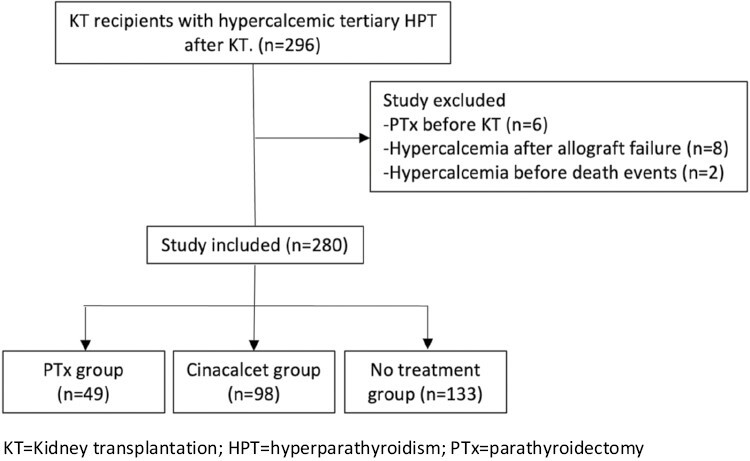
Inclusion and exclusion criteria. KT = kidney transplantation; HPT = hyperparathyroidism; PTx = parathyroidectomy.

We reviewed all KT recipients from 2015 to 2019 diagnosed with hypercalcemic tertiary hyperparathyroidism (HPT) post-KT at the University of Alabama at Birmingham (*n* = 296). KT recipients were identified from our institutional kidney transplant database. Hypercalcemic tertiary HPT was defined as elevated PTH levels with hypercalcemia post-KT. All included patients had either elevated PTH levels from the time of KT until December 2021 or received treatment with either cinacalcet or PTx. Included patients had at least 2 elevated serum calcium levels post-KT. We excluded recipients who underwent PTx before KT (*n* = 6), recipients who had first elevated serum calcium after graft failure (*n* = 8), and recipients who developed hypercalcemia before death events (*n* = 2) (Fig. 1).

Patients were categorized by treatment group. Recipients who did not receive PTx or cinacalcet post-KT were classified as the no treatment group. At our institution, cinacalcet is often considered the first choice of treatment when KT recipients develop hypercalcemic tHPT. When KT recipients do not tolerate or cannot afford cinacalcet or hypercalcemic tHPT is not controlled by cinacalcet, KT recipients are referred for PTX. Recipients who only received cinacalcet post-KT were classified as the cinacalcet group. Of note, two recipients who received cinacalcet for only 5 and 7 days, respectively, from the date of first hypercalcemia to the date of graft failure were classified to the no treatment group. Recipients who received parathyroidectomy post-KT were classified as the PTx group. Our aim was to 1) evaluate the characteristics of patient demographics and kidney transplantation in each treatment group, 2) assess the characteristics of HPT post-KT for each treatment group, and 3) investigate the association between different treatment modalities and allograft survival.

Data regarding recipient and donor demographics, allograft survival of KT, and severity of hypercalcemic tertiary HPT were collected from electronic medical records. Charlson Comorbidity Index was calculated to reflect baseline comorbidity in each group.^33^ Serum calcium was adjusted when serum albumin was less than 4.0 g/dL using the formula: corrected calcium = [0.8*(4 − serum albumin) + serum calcium]. Delayed graft function (DGF) was defined as a need for dialysis within 7 days post-KT. All-cause graft failure was defined as requiring long-term renal replacement therapy post-KT or death. Death-censored graft failure was defined as a return to chronic dialysis, censoring for death.

Descriptive statistics were performed and tested for skewness. Continuous data with normal distributions were described as mean ± standard deviation (SD), while those with non-normal distributions were described as the median and interquartile range (IQR). Bivariate analyses were conducted using analysis of variance (ANOVA), Kruskal-Wallis H, and Chi-squared tests. Post hoc analyses were performed to compare every 2 groups with adjusted *P* values.

The association between treatment modality and allograft survival was evaluated using time-varying Cox regression. While the index date for all subjects was the date of KT, this study incorporated a delayed entry at the time of first hypercalcemia to account for the variation in time at which a recipient would first be considered for treatment post-KT. For example, an individual whose first elevated calcium level was recorded at 5 months post-KT would begin contributing time at risk at month 5 post-KT. The endpoint was the date of graft failure, death, or last follow-up. The intervention (no treatment vs. cinacalcet vs. PTx) was used in Cox regression models as a time-fixed and time-varying covariate. When the intervention was considered as a time-varying covariate, 2 time variables were created, including the interval between entry time and the time of intervention and the interval between the time of intervention and endpoint. Other covariates in the Cox regression model were chose based on clinical significant and statistical results from the bivariate analyses.

### Sensitivity Analysis

Fourteen recipients in the PTx group received cinacalcet before PTx and did not require cinacalcet after PTX and one recipient required cinacalcet permanently due to persistent tHPT after PTx were excluded from the sensitivity analysis. Sensitivity analyses of time-varying Cox regression were conducted to compare death-censored and all-cause allograft failure by treatment groups.

IBM SPSS version 29.0 and SAS 9.4 were used for data analysis. Variables were considered statistically significant at *P* < .05. This study was approved by the UAB Institutional Review Board.

## Results

Of 280 KT recipients with hypercalcemic tertiary HPT included in this study, 49 recipients underwent parathyroidectomy, 98 recipients were only prescribed cinacalcet, and 133 recipients did not receive any treatment. Recipients who received treatment were more likely to require dialysis before KT (*P* = .019) and had a longer dialysis vintage (ie, length of time on dialysis) (*P* = .017) than the no treatment group. We did not find differences in the patient’s demographics, baseline comorbidities, insurance type, or etiology of ESKD between groups (Table 1). There was no difference in the characteristics of kidney transplantation between the 3 groups (Table 2). There was significantly more death (*P* = .003), death-censored graft failure (*P* < .001), and all-cause graft failure (*P* < .001) in the no treatment group than in the treatment groups.

**Table 1.** Patient demographics.

	PTx group (*n* = 49)	Cinacalcet group (*n* = 98)	No treatment group (*n* = 133)	Total (*n* = 280)	*P*-value
Age (years, mean ± SD)	50 ± 10	51 ± 12	52 ± 11	51 ± 11	.319
Sex (%)					.361
Female	17 (34.7%)	31 (31.6%)	54 (40.6%)	102 (36.4%)	
Male	32 (65.3%)	67 (68.4%)	79 (59.4%)	178 (63.3%)	
Race/ethnicity (%)					.268
Black	28 (57.1%)	74 (75.5%)	87 (65.4%)	189 (67.5%)	
White	21 (42.9%)	24 (24.5%)	44 (33.1%)	89 (31.8%)	
Asian	0	0	1 (0.8%)	1 (0.4%)	
Hispanic	0	0	1 (0.8%)	1 (0.4%)	
Charleson Comorbidity Index median (IQR)	4 (3-6)	4 (3-5)	4 (3-6)	4 (3-6)	.099
Primary Insurance at KT					.299
Medicare	35 (71.4%)	75 (76.5%)	99 (74.4%)	209 (74.9%)	
Private	9 (18.4%)	16 (16.3%)	30 (22.6%)	55 (19.7%)	
Medicaid	5 (10.2%)	6 (6.1%)	4 (3.0%)	15 (5.4%)	
Second Insurance at KT	30 (61.2%)	60 (61.2%)	89 (66.9%)	179 (63.9%)	.612
Etiology of ESKD					.268
Hypertension	20 (40.8%)	41 (41.8%)	44 (33.1%)	105 (37.5%)	
Diabetes	9 (18.4%)	22 (22.4%)	41 (31.6%)	73 (26.1%)	
Glomeronephropathy	6 (12.2%)	13 (13.3%)	18 (13.5%)	37 (13.2%)	
Polycystic kidney Disease	5 (10.2%)	10 (10.2%)	5 (3.8%)	20 (7.1%)	
Other	9 (18.4%)	12 (12.2%)	24 (18.0%)	45 (16.1%)	
Dialysis before KT (Yes)	48 (98.0%)	98 (100.0%)	124 (93.2%)	270 (96.4%)	.019
Dialysis vintage (months, mean ± SD)	78.5 ± 44.5	77.5 ± 35.8	63.5 ± 44.9	71.3 ± 42.4	.017

Abbreviation: ESKD = end stage kidney disease.

**Table 2.** Characteristics and clinical outcome of kidney transplantation.

	PTx group (*n* = 49)	Cinacalcet group (*n* = 98)	No treatment group (*n* = 133)	Total (*n* = 280)	*P*-value
Donor type					.638
Deceased	111 (83.5%)	86 (87.8%)	111 (83.5%)	238 (85.0%)	
Living	22 (16.5%)	12 (12.2%)	22 (16.5%)	42 (15.0%)	
Donor age	34 ± 12	36 ± 16	39 ± 13	37 ± 14	.123
KDPI (%), median (IQR)	30.0 (20.0-62.0)	37.5 (23.0-67.5)	55.0 (33.5-70.5)	51.0 (23.0-68.0)	.080
% PRA I, median (IQR)	0 (0-3)	0 (0-10)	0 (0-23.3)	0 (0-14)	.745
% PRA II, median (IQR)	0 (0-3)	0 (0-3)	0 (0-3)	0 (0-3)	.750
Shared antigen (n/10)	3.0 ± 2.0	2.2 ± 1.6	3.0 ± 2.0	2.7 ± 1.9	.013
Anastomosis					.782
UC	43 (87.8%)	87 (88.8%)	114 (85.7%)	244 (87.1%)	
UU	6 (12.2%)	11 (11.2%)	19 (14.3%)	36 (12.9%)	
Induction immunosuppression					.074
Thymoglobulin	36 (73.5%)	70 (71.4%)	95 (71.4%)	201 (71.8&)	
Basiliximab	1 (2.0%)	7 (7.1%)	19 (14.3%)	27 (9.6%)	
Alemtuzumab	11 (22.4%)	21 (21.4%)	17 (12.8%)	49 (17.5%)	
Thymoglobulin and Basiliximab	1 (2.0%)	0	2 (1.5%)	3 (1.1%)	
Maintenance immunosuppression					
Tacrolimus	49 (100%)	98 (100%)	133 (100%)		—
Mycophenolate	48 (98.0%)	98 (100%)	131 (98.5%)	277 (98.9%)	.326
Prednisone	40 (81.6%)	78 (79.6%)	116 (87.2%)	234 (83.6%)	.276
DGF	3 (6.1%)	13 (13.3%)	22 (16.5%)	38 (13.6%)	.190
Rejection before discharge	0	0	1 (0.8%)	1 (0.4%)	.574
Rejection 6 months post-KT	0	0	1 (0.8%)	1 (0.4%)	.165
Rejection 1 yr post-RTx	0	0	1 (0.8%)	1 (0.4%)	.868
Death (%)	4 (8.2%)	15 (15.3%)	38 (28.6%)	57 (20.4%)	.003
Death-censored graft failure (%)	2 (4.1%)	3 (3.1%)	22 (16.5%)	27 (9.6%)	<.001
All-cause graft failure (%)	6 (12.2%)	18 (18.4%)	53 (39.8%)	77 (27.5%)	<.001
PTH on admission for KT, pg/mL, median (IQR)	718 (324-953)	511 (311-944)	401 (257-644)	472 (282-773)	.002
Serum calcium on admission for KT, mg/dL (mean ± SD)	9.7 ± 0.9	9.3 ± 0.9	9.4 ± 0.9	9.4 ± 0.9	.077
Hypercalcemia on admission for KT	10 (20.4%)	9 (9.2%)	10 (7.5%)	29 (10.4%)	.036
Cinacalcet use on admission for KT	39 (79.6%)	70 (71.4%)	64 (48.1%)	173 (61.8%)	<.001
Time from KT to first hypercalcemia, months Median (IQR)	0 (0-1)	0 (0-2)	2.0 (0-6)	1 (0-4)	<.001
Most recent PTH pg/mL, median (IQR)	147 (80-273)	207 (148-310)	169 (124-306)	183 (126-306)	.012
Most recent serum calcium mg/dL, (mean ± SD)	9.1 ± 0.8	9.8 ± 0.7	9.7 ± 0.8	9.6 ± 0.8	<.001

Abbreviations: DGF = delayed graft function; KDPI = Kidney Donor Profile Index; %PRA = percent panel reactive antibody; UC = Ureterovesical anastomosis, UU = Ureteroureterostomy.

We then evaluated the characteristics of HPT post-KT in each treatment group (Table 2). Compared to the no treatment group, recipients who were treated for hypercalcemic tHPT had higher PTH levels before KT (*P* = .002) and earlier onset of hypercalcemia post-KT (*P* < .001). The mean (±SD) follow-up length for all patients was 43.5 ± 19.4 months. At the most recent follow-up, there were statistically significant differences in PTH (*P* = .012) and serum calcium (*P* < .001) levels between each group. After conducting post hoc analyses, the PTx group had a significantly lower median (IQR) PTH levels than the cinacalcet group (adjusted *P* = .015). There was no difference in most-recent PTH levels between the PTx group and the no treatment group (adjusted *P* = .643) or cinacalcet group vs. no treatment group (adjusted *P* = .100). The PTx group had significantly lower medium (±SD) serum calcium than the cinacalcet group (adjusted *P* < .001) and the no treatment group (adjusted *P* < .001). There was no difference in serum calcium between the cinacalcet group and the no treatment group (adjusted *P* = .215).

We further investigated the association of HPT treatment post-KT and allograft outcome. When treatment groups were regarded as time-fixed covariates, only treatment groups was independently associated with death-censored graft failure after adjusting for age at KT, race, time from KT to hypercalcemia, treatment for hypercalcemic tHPT, DGF, dialysis condition before KT, donor type, and insurance at KT (Table 3). Given the small number of death-censored graft failure events (*n* = 27), we only included sex and treatment groups in the late-entry time-varying Cox regression model. Compared to the no treatment group, recipients who were treated with cinacalcet (HR .14, 95% CI 0.004-0.47, *P* = .002) or PTx (HR 0.18, 95% CI 0.04-0.76, *P* = .02) had a lower risk of death-censored graft failure (Table 4). The statistical results remained in the sensitivity analysis after excluding recipients who were treated with cinacalcet and then PTx. We then changed the reference group from the treatment group covariate from the no treatment group to the cinacalcet group to compare allograft survival between the cinacalcet group and the PTx group; we did not find difference between the cinacalcet and PTx groups

**Table 3.** Cox regression models for death-censored graft failure by adjusting treatment as time-fixed covariates.

Covariates	HR	95% CI	*P*-value
Age at KT	0.977	0.944-1.011	.184
Race (Black vs. non-Black)	1.403	0.593-3.321	.441
Time from KT to hypercalcemia, months	1.012	0.951-1.078	.699
Treatment group (no treatment group as reference)			
Cinacalcet group	0.142	0.042-0.474	.002
PTx group	0.150	0.035-0.642	.011
DGF (yes vs. no)	2.297	0.926-5.701	.073
Dialysis before KT (no vs. yes)	1.139	0.154-8.407	.899
Dialysis vintage	0.998	0.989-1.006	.592
Donor type (deceased vs. living)	1.544	0.465-5.128	.478
Primary insurance at RTx (private as reference)			
Medicare vs. private	1.816	0.542-6.093	.334
Medicaid vs. private	2.547	0.512-12.661	.253
Secondary Insurance at KT (yes vs. no)	0.990	0.444-2.208	.981

**Table 4.** Cox regression models for death-censored graft failure by adjusting treatment as time-varying covariates.

Variable	HR	95%CI	*P*-value
Treatment group (no treatment group as reference)			
Cinacalcet group	0.14	0.04-0.47	.002
PTx group	0.18	0.04-0.76	.02
Sex (male vs. female)	1.84	0.77-4.39	.17

A Cox regression model with the same covariates was performed using treatment groups as fixed covariates to investigate all-cause graft failure (Table 5). Treatment group, age at KT, DGF, and primary insurance were independently associated with all-cause graft failure. Given more all-cause graft failure events (*n* = 77) than death-censored graft failure events, we included treatment groups, age at KT, sex, race, donor type, dialysis before KT, and insurance at KT as covariates in the late-entry time-varying Cox regression model for all-cause graft failure (Table 6). Treatment of hypercalcemic tHPT, age at KT, and Medicare as primary insurance were independently associated with all-cause graft failure. Compared to no treatment group, recipients who were treated with cinacalcet (HR 0.386, 95% CI 0.224-0.664, *P* < .01) or PTx (HR 0.282, 95% CI 0.120-0.661, *P* < .001) had a lower risk of all-cause graft failure. We did not find any significance between the cinacalcet and PTx groups. The statistical results remained in the sensitivity analysis after excluding recipients who were treated with cinacalcet and then PTx.

**Table 5.** Cox regression models for all-cause graft failure by adjusting treatment as time-fixed covariates.

Variable	HR	95% CI	*P*-value
Age at KT	1.031	1.009-1.053	.005
Sex (female vs. male)	1.398	0.890-2.196	.146
Race (Black vs. non-Black)	1.392	0.836-2.317	.204
Time from KT to hypercalcemia, months	1.003	0.964-1.044	.866
Treatment group (no treatment group as reference)			
Cinacalcet group	0.375	0.219-0.641	<.001
PTx group	0.205	0.088-0.479	<.001
DGF (yes vs. no)	2.198	1.282-3.770	.004
Dialysis before KT (no vs. yes)	1.317	0.323-5.368	.701
Dialysis length before KT	0.998	0.993-1.004	.530
Donor type (deceased vs. living)	2.243	0.975-5.160	.058
Primary Insurance at KT (private as reference)			
Medicare	2.532	1.162-5.517	.019
Medicaid	1.213	0.313-4.696	.780
Secondary Insurance at KT (Yes vs. No)	0.895	0.562-1.426	.641

**Table 6.** Cox regression models for all-cause graft failure by adjusting treatment as time-varying covariates.

Variable	HR	95%CI	*P*-value
Treatment group (no treatment as ref)			
Cinacalcet group	0.386	0.224-0.664	<.001
PTx group	0.282	0.120-0.661	<.001
Age at transplant	1.024	1.003-1.046	.023
Gender (male vs. female)	0.779	0.482-1.257	.31
Race (AAF/B vs. non-AAF/B)	1.208	0.707-2.063	.49
Donor type (deceased vs. living)	1.770	0.823-3.804	.14
Dialysis before transplant (yes vs. no)	1.25	0.32-4.83	.75
Primary insurance (private as ref.)			
Medicare vs. private	2.954	1.143-5.887	.02
Medicaid vs. private	1.421	0.365-5.529	.61
Secondary Insurance (yes vs. no)	0.69	0.41-1.17	.17

## Discussion

To our knowledge, this is the first study to leverage time-varying survival analyses to overcome survivor treatment selection bias in treatment of tertiary HPT, and to clearly demonstrate an association between treatment of tertiary HPT and improved allograft survival. HPT is often managed by oncologic surgeons and endocrine surgeons.^34^ This study offers evidence supporting the treatment of hypercalcemic hyperparathyroidism after kidney transplantation. Specifically, in this retrospective study of 280 KT recipients who developed hypercalcemic tertiary HPT demonstrated compared to no treatment group, recipients who underwent PTx or were prescribed cinacalcet had lower risks of death-censored allograft failure and all-cause allograft failure. With an exponential increase in the need for KT worldwide and vastly insufficient kidney donors,^35^ HPT should be screened and treated following KT to protect a finite public health resource.

The association of persistent HPT post-KT and allograft failure has been reported in several retrospective studies.^16,36-39^ A longitudinal follow-up of 911 KT recipients having hypercalcemic tertiary HPT 1 year post-KT showed that hypercalcemic tertiary HPT was associated with an increased risk of death-censored graft failure. Crepeau et al.^39^ reviewed 824 KT recipients with elevated PTH levels post-KT with hypercalcemia and normocalcemia. They concluded that persistent HPT at 1-year post-KT had a 1.37-fold higher risk of all-cause graft loss and a 1.6-fold higher risk of death-censored graft loss. This is consistent with our results that no treatment of hypercalcemic tertiary HPT was associated with worse allograft survival. The possible mechanism for a causative role of tertiary HPT for allograft dysfunction and mortality includes the promotion of vascular calcification^40^ and renal interstitial fibrosis.^41^

Despite the clinical consequence, tertiary HPT in KT recipients are undertreated. In a large cohort study of 30,127 Medicare-ensured KT recipients, only one-fifth of the cohorts were treated with PTx and/or cinacalcet.^14^ In this study, half of our cohort (47.5%) did not receive any treatment for hypercalcemic tertiary HPT. One potential explanation for this undertreatment is the concern about the adverse effect of treatment on allograft function. The effect of PTx or cinacalcet on allograft function is controversial. Kruse et al. reported a statistically significant increase in serum creatinine at months 2 and 3 after cinacalcet initiation in a prospective study of 14 cohorts.^27^ Patecki et al. retrospectively reviewed 47 KT recipients who underwent PTx post-KT and reported a median annual estimated glomerular filtration rate (eGFR) change of −0.5 mL/minute before and + 1.0 mL/minute post-PTx.^26^ Okada et al.^42^ compared 55 and 53 patients who underwent PTx before and post-KT and found a more significant decrease in the eGFR 12-36 months after PTx post-KT. The decline in allograft function was cited as a rationale for favoring PTx before KT in the KDIGO guideline.^9^ However, the above studies are often from single institutions and were subject to a selection bias. Frey et al.^43^ performed a meta-analysis of 7 studies assessing the evolution of graft function post-PTx or cinacalcet introduction. The meta-analysis shows that PTx and cinacalcet do not significantly impair graft function in KT recipients. Moreover, the decrease in allograft function is often resolved in long-term follow-up. A previous study from our institution compared KT recipients who underwent PTx pre- and post-KT with a median follow-up length of 58 months; no significant changes in allograft function were found in either group.^8^ van der Plas^29^ also reported a similar eGFR in the pre-KT and post-KT groups at 5 years after transplantation. The fluctuation of the allograft function is multifactorial. We used allograft failure as a dependent outcome in our study and no treatment group as the control group to adjust the confounding effect of hypercalcemic tertiary HPT on allograft function. We found that recipients who were treated for hypercalcemic tertiary HPT were less likely to develop allograft failure. This finding demonstrates the importance of managing HPT in KT recipients to protect allograft function.

PTx is the treatment of choice for tHPT.^12^ However, cinacalcet has been used off-label for treating HPT in KT recipients. Although several studies have shown that cinacalcet effectively controlled hypercalcemia post-KT,^27,44^ cinacalcet has failed to benefit bone mineralization,^9^ and its long-term effect is unknown. Several studies have compared the clinical outcomes of PTx and cinacalcet among KT recipients. Cruzado et al. conducted a randomized control trial of 15 patients with PTx and 15 with cinacalcet.^45^ Patients who underwent PTx were more likely to achieve normocalcemia, had a greater reduction of PTH and a significant increase in femoral neck bone mineral density at 12 months. Soliman et al.^46^ performed a prospective open-label controlled study to compare the clinical outcomes of PTx and cinacalcet in KT recipients. Sixteen patients undergoing PTx and 45 patients with cinacalcet were followed up for 1 year. At 12 months, the 2 groups had no difference in serum calcium, PTH, and serum creatinine. Finnerty et al.^31^ retrospectively reviewed 133 KT recipients with tertiary HPT (33 received PTx and 100 received cinacalcet) and compared allograft outcomes between the 2 groups. They found that allograft survival was improved in the PTx cohorts compared to the cinacalcet-alone group. Given the differing time of treatment initiation and potential for survivor treatment selection bias, this finding should be cautiously interpreted.^32^ KT recipients who live longer could have more opportunities to select PTx. Moreover, it is unclear if their Cox regression analysis model included any confounding covariates for adjustment, which can cause an omitted variable bias. A strength of our study is the use of delayed entry time-varying Cox regression was used to mitigate the survivor treatment selection bias and adjust confounding effects associated with graft failure. Our study found that KT recipients who were treated for hypercalcemic tertiary hyperparathyroidism (PTx or cinacalcet) were associated with lower risks of allograft failure. We did not find a difference in allograft survival between the PTx and cinacalcet groups. However, 14 (28.5%) recipients failed cinacalcet before the referral for PTx. The time from KT to PTx was significantly longer than from KT to cinacalcet initiation. Perhaps KT recipients in the PTx group had more severe HPT than the cinacalcet group.

This study has several limitations, including those inherent to any retrospective study. The 3 groups are not homogenous in terms of HPT. The untreated group had milder diseases, as reflected by lower PTH levels at KT and later onset of hypercalcemia post-KT compared to the treated groups. However, the untreated group had a worse outcome of allograft survival compared to the treated groups. This further demonstrated the positive effect of treating HPT on allograft survival. The generalizability of study results is limited due to small sample size and single-institution cohorts. The number of graft failure events was relatively small, which limited the covariates adjusted in the Cox regression models. Given that this is a retrospective study, we cannot conclude causality between the treatment of hypercalcemic tertiary HPT and graft outcome. However, we used adjusted delayed entry time-varying Cox regression models to minimize confounding effects. Our findings stress the need for large randomized controlled trials with long-term follow-ups to investigate the causality between tertiary HPT treatment and allograft outcome.

In conclusion, this study demonstrates that treatment of hypercalcemic tertiary hyperparathyroidism post-kidney transplantation was associated with improved allograft survival. To achieve this survival benefit, hypercalcemic hyperparathyroidism should be screened and treated following kidney transplantation.

## Data Availability

The data underlying this article cannot be shared publicly for the privacy of individuals that participated in the study. The data will be shared on reasonable request to the corresponding author.
